# Soil nitrogen availability and microbial carbon use efficiency are dependent more on chemical fertilization than winter drought in a maize–soybean rotation system

**DOI:** 10.3389/fmicb.2024.1304985

**Published:** 2024-03-14

**Authors:** Wenqing Bao, Peng He, Lin Han, Xiaowei Wei, Lei Feng, Jianqin Zhu, Jihua Wang, Xuechen Yang, Lu-Jun Li

**Affiliations:** ^1^School of Life Science and Technology, Harbin Normal University, Harbin, China; ^2^Hailun National Observation and Research Station of Agroecosystems, State Key Laboratory of Black Soils Conservation and Utilization, Northeast Institute of Geography and Agroecology, Chinese Academy of Sciences, Harbin, China; ^3^Jilin Provincial Key Laboratory for Plant Resources Science and Green Production, Jilin Normal University, Siping, China; ^4^College of Advanced Agricultural Sciences, University of Chinese Academy of Sciences, Beijing, China

**Keywords:** ammonium availability, Mollisol cropland, nitrate availability, phospholipid fatty acid analysis, snow removal, soil microbial characteristics

## Abstract

Soil nitrogen (N) availability is one of the limiting factors of crop productivity, and it is strongly influenced by global change and agricultural management practices. However, very few studies have assessed how the winter drought affected soil N availability during the subsequent growing season under chemical fertilization. We conducted a field investigation involving snow removal to simulate winter drought conditions in a Mollisol cropland in Northeast China as part of a 6-year fertilization experiment, and we examined soil physicochemical properties, microbial characteristics, and N availability. Our results demonstrated that chemical fertilization significantly increased soil ammonium and total N availability by 42.9 and 90.3%, respectively; a combined winter drought and fertilization treatment exhibited the highest soil N availability at the end of the growing season. As the growing season continued, the variation in soil N availability was explained more by fertilization than by winter drought. The Mantel test further indicated that soil Olsen-P content and microbial carbon use efficiency (CUE) were significantly related to soil ammonium availability. A microbial community structure explained the largest fraction of the variation in soil nitrate availability. Microbial CUE showed the strongest correlation with soil N availability, followed by soil available C:P and bacteria:fungi ratios under winter drought and chemical fertilization conditions. Overall, we clarified that, despite the weak effect of the winter drought on soil N availability, it cannot be ignored. Our study also identified the important role of soil microorganisms in soil N transformations, even in seasonally snow-covered northern croplands.

## 1 Introduction

Approximately one-quarter of the world’s arable land is located in snowy and cold regions, which are experiencing more intense and frequent drought stress in the context of global change (Zhou et al., 2017). Winter drought would result in a reduction in snow cover, and soils would be increasingly exposed to cold air, further exacerbating soil freezing, especially during cold spells (Vankoughnett and Henry, 2014). Soil frozen can alter the diversity of soil organisms and biogeochemical cycling in terrestrial ecosystems, thereby affecting the nutrient supply and growth of plants during the subsequent growing season (Makoto et al., 2014; Urakawa et al., 2014).

Soil nitrogen (N) availability is a critical parameter controlling organisms’ growth and ecosystem functions, but it can easily be reduced through leaching or trace gas emissions (Urakawa et al., 2014). A previous meta-analysis revealed that the winter drought reduced the availability of N in the soils and increased the potential nutrient leaching, owing to the cell burst of soil microorganisms and plant roots, which reduced the activities of soil microorganism and plant roots (Song et al., 2017). In order to mitigate the negative effects of extreme climate events on soil quality and reduce available N losses, effective management practices are urgently required to increase soil fertility and N availability. Chemical fertilizer supply is an effective strategy to enhance crop yields and improve soil fertility worldwide (Nguyen et al., 2018), and its large-scale use is also associated with massive disruptions to global biodiversity and N and phosphorus (P) cycles. However, we are still far from fully understanding the responses of soil N availability to the combined effects of the predicted changes in snowfall (e.g., winter drought) and fertilization.

Chemical fertilizer, such as ammonium hydrogen phosphate, as an available N source can directly increase soil N availability. A 28-year maize–soybean rotation experiment showed that, compared to no fertilization, soil available N increased by 18.7% in chemical fertilization (Zhang et al., 2023). However, the dissolved inorganic N (DIN:NH_4_^+^-N and NO_3_^−^-N) of fluvo-aquic soil was unchanged after 2 years of chemical fertilization (Zhao et al., 2019). Previous findings indicated that the effect of chemical fertilization on soil N availability exhibited a time-scale dependence. Chemical fertilization can also affect soil N availability indirectly via altered soil N transformation rates catalyzed by plants and microorganisms (Gentile et al., 2008; Hu et al., 2022). For example, in urea fertilization soils, the release of organic compounds into the soil can significantly alter microbial metabolism and activity in its immediate surroundings, thus enhancing organic N mineralization (Wiesenbauer et al., 2024). In addition, soil N availability was reported to be increased in response to fertilization in crop drought soils, whereas other studies observed decreased N availability in grassland drought soils (Hartmann et al., 2013; Gelfand et al., 2015). The effect of fertilization on soil N availability may be modified by the effect of winter drought, and we have limited knowledge regarding the potential key factors associated with soil N availability.

Soil N availability has been found to exhibit significant relationships with multiple abiotic (e.g., moisture and temperature) and biotic (i.e., plants and soil microorganisms) factors (Chaves et al., 2021; Liu et al., 2023). There are many pathways through which soil microorganisms can affect soil N availability. Microbial carbon (C) use efficiency (CUE) serves as an integrative index to describe the balance of soil nutrients (Tao et al., 2023). It has been reported that a decrease in soil N availability was accompanied by an increase in microbial CUE (Silva-Sánchez et al., 2019). A high microbial CUE might cause soil microorganisms to allocate more C to growth, thereby resulting in a lower metabolic cost of soil N acquisition (Manzoni et al., 2012). Additionally, soil N availability often depends on the abundance of N-cycling microbial functional genes (Hu et al., 2022), such as those of ammonia-oxidizing archaea and bacteria, which can alter N mineralization rates, thereby positively affecting soil N availability (Okano et al., 2004). Yu et al. (2018) have reported that the increased microbial biomass but not altered microbial community structure was beneficial to soil N transformation in crop rhizosphere. Although soil microorganisms have the potential to be a strong predictor linked to soil N availability, the influence direction is ambiguous. Moreover, the altered stoichiometry of soil substrates by winter drought and chemical fertilization could also affect microbial characteristics and soil N availability (Li et al., 2021). There are open questions about the relationships between soil properties and microorganisms and their effects on soil N availability.

Herein, we used snow removal to simulate winter drought and added chemical fertilizers to soils during spring sowing in a maize–soybean rotation cropland in the Mollisols area in Northeast China. We evaluated the responses of soil ammonium, nitrate, and total N availability to the effects of winter drought and chemical fertilization during the subsequent growing season. To identify potential factors related to soil N availability, we investigated the soil physicochemical properties (e.g., soil water content, pH, dissolved organic carbon (DOC), DIN, Olsen-P, and available potassium (AK) content) and microbial characteristics (e.g., biomass, community structure, CUE, and extracellular enzyme activities). We hypothesized that (1) soil N availability would increase in response to chemical fertilization, but it would decrease in response to winter drought because decreases in soil temperature could reduce the activities of soil microorganisms (Song et al., 2017), which were responsible for N mineralization (Mooshammer et al., 2014) and (2) microbial characteristics, rather than soil properties, would be the primary factor associated with soil N availability, because soil microorganisms were sensitive to changes in temperature, especially in cold regions.

## 2 Materials and methods

### 2.1 Site description

The experiment was conducted at the Hailun National Observation and Research Station of Agroecosystems of the Chinese Academy of Science (47°26′N, 126°38′E, 240 m in elevation), which is located in the central region of the Mollisols area in Northeast China. The study area exhibits a typical temperate continental monsoon climate with cold winters and hot summers. The mean annual air temperature is 2.0°C (which has been the same in the past 70 years). The non-growing season lasts from October to April with the lowest mean monthly temperature of −23°C in January, whereas the growing season lasts from May to September with the highest mean monthly temperature of 21°C in July (Hao et al., 2022). The mean annual precipitation is 550 mm, and more than 65% of precipitation is concentrated from June to August. Snowfall generally begins in early to mid-November, with snowmelt occurring by late March or early April. The soil type is typical Mollisol (USDA soil taxonomy) with a silty clay loam texture. The soil had a pH of 5.1, a total carbon (TC) content of 30.8 g kg^−1^, a total nitrogen (TN) content of 2.3 g kg^−1^, a total phosphorus (TP) content of 1.0 g kg^−1^, and a total potassium (TK) content of 22.6 g kg^−1^ before the start of the experiment in 2017.

### 2.2 Experimental design

The experiment was initiated in June 2017 with a completely randomized design. We established 12 plots (each has an area of 2.5 m × 2.8 m) and separated plots with a 0.5-m tall white steel plate (0.2 m aboveground, 0.3 m belowground). There were four treatments, namely a non-drought and non-fertilization (control: C), a winter drought (D), a chemical fertilization (F), and a combined winter drought and chemical fertilization (DF), which resulted in three replicates. Each plot was divided into four ridges, with a length and a width of 2.5 and 0.7 m, respectively. Maize (*Zea mays* L.) was planted in rotation with soybean (*Glycine max* Merr.) in alternate years. In 2017, maize was planted, and in the following year, soybeans were planted. The planting density of maize and soybean was 12 plants per ridge and 24 plants per ridge, respectively.

For the fertilization treatments, the chemical fertilizers, such as urea, ammonium hydrogen phosphate, and potassium sulfate, were applied at the rates of 138 kg N, 70 kg P_2_O_5_, and 20 kg K_2_O ha^−1^ y^−1^ for maize, and 64 kg N, 70 kg P_2_O_5_, and 20 kg K_2_O ha^−1^ y^−1^ for soybean (Zhang et al., 2023). For maize planting years, P and kalium (K) fertilizers were applied during the sowing period, and N fertilizer was applied at the sowing and jointing stages with a ratio of 1:2. For soybean planting years, all chemical fertilizers were applied during the sowing period.

In late October 2021, we set up a 1-year winter drought (snow removal) treatment. We attached a black plastic net (1 cm^−2^ in hole size) to the soil in each plot. To prevent snow accumulation on the snow removal treatments, we used brooms to remove the snow out of the plot down to the level of the plastic net (typically following major snowfall events). Following the final snowmelt, we removed the plastic nets when the snow was completely melted.

Soil temperature and soil water content (SWC) were measured using sensors (MEC10, Zheqin Technology Co., Ltd., Dalian, Liaoning, China) at a depth of 0–5 cm. The measurements were taken continuously from November 2021 to October 2022.

### 2.3 Soil sample collection

In May and October 2022, we collected five soil cores (3.5 cm in diameter, 0–10 cm depth) randomly from each plot and homogenized them into a composite soil sample. All soil samples were sieved through a 2-mm mesh to remove roots, litter, debris, and stones. The soil samples were divided into three parts, with one part stored at 4°C for measuring SWC, DOC, NH_4_^+^-N, NO_3_^−^-N, soil extracellular enzyme activity (EEA), and microbial biomass; one part stored at −20°C for phospholipid fatty acid (PLFA) analysis; and the last part air-dried to determine soil pH, Olsen-P, and AK contents.

### 2.4 Soil properties measurement

SWC was measured after drying 10 g of fresh soil at 105°C until a constant weight was achieved. Soil pH was measured by shaking a 1:2.5 ratio of air-dried soil to deionized water for 30 min. DOC was extracted by 0.5 M K_2_SO_4_ and then measured using a TOC analyzer (enviro TOC, Elementar, Germany). Soil NH_4_^+^-N and NO_3_^−^-N were extracted by 2 M KCl and analyzed by a continuous flow analyzer (AA3, Seal Analytical, Norderstedt, Germany). Olsen-P was determined colorimetrically (malachite green) after extracting samples with 0.5 M NaHCO_3_. AK was digested with 1 M ammonium acetate and detected by flame photometry.

### 2.5 Soil enzyme activity

We quantified soil EEAs using a modified fluorometric technique (Yang et al., 2023), including labile C-cycling enzymes: α-1,4-glucosidase (AG), β-1,4-glucosidase (BG), β-xylosidase (BX), and β-D-cellobiosidase (CBH), N-cycling enzymes: β-1,4-N-acetylglucosaminidase (NAG) and L-leucine aminopeptidase (LAP), and P-cycling enzymes: acid phosphatase (AP). The corresponding substrates of these EEAs were as described in Matulich et al. (2015). Soil suspensions were prepared by adding 1 g of fresh soil to 100 mL of 50 mM acetate buffer. Four replicates were used for sample assay wells, blank wells, quench standard wells, negative control wells, and reference standard wells. The 96-deep-well microplates were covered and incubated in the dark at 20°C for 4 h, and the reaction was stopped by adding 10 μL of 1 M NaOH to each well. Fluorescence was measured using a microplate reader (Synergy LX, BioTek Instruments, Winooski, VT, United States) with 360 nm excitation and 460 nm emission filters.

### 2.6 Microbial biomass and carbon use efficiency

Microbial biomass carbon (MBC), nitrogen (MBN), and phosphorus (MBP) were determined with the chloroform fumigation-extraction method (Joergensen, 1996). Microbial CUE derived from the biogeochemical equilibrium model was calculated as follows (Sinsabaugh and Follstad Shah, 2012):


$$\mathrm{CUE}={\mathrm{CUE}}_{\text{max}}\times {\left\{\frac{S_{C:N}\times S_{C:P}}{\left(K_{C:N}+S_{C:N}\right)\times \left(K_{C:P}+S_{C:P}\right)}\right\}}^{0.5}$$

$$S_{C:N}=\frac{B_{C:N}}{L_{C:N}\times {EEA}_{C:N}}$$

$$S_{C:P}=\frac{B_{C:P}}{L_{C:P}\times {EEA}_{C:P}}\text{,}$$

where *K_C:N_* and *K_C:P_* represent the half-saturation constants for CUE based on the availability of C, N, and P. We assumed that *K_C:N_* and *K_C:P_* were 0.5 for the model and CUE_max_ was 0.6 in this study (Sinsabaugh et al., 2016). *EEA_C:N_* was calculated as (AG + BG + BX + CBH)/(NAG + LAP), and *EEA_C:P_* was calculated as (AG + BG + BX + CBH)/AP. *B_C:N_* and *B_C:P_* were the elemental C:N and C:P ratios of microbial biomass. *L_C:N_* and *L_C:P_* were estimated as DOC:DIN and DOC:Olsen-P, respectively.

### 2.7 Phospholipid fatty acid analysis

PLFAs were extracted from the soils as described by Yang et al. (2020). The 19:00 was used as an internal standard. The fatty acids (FAs) 14:00, 15:00, 16:00, 17:00, and 18:00 were chosen to represent the non-specific bacteria (Frostegård and Bååth, 1996; Leckie, 2005); i14:0, a15:0, i15:0, i15:1ω6c, i16:0, a17:0, and i17:0 were used to represent the Gram-positive bacteria (G^+^); 16:0 2OH, 16:1ω7c, 16:1ω9c, cy17:0ω7c, 17:1ω8c, 18:1ω5c, 18:1ω6c, 18:1ω7c, and cy19:0ω7c were chosen to represent the Gram-negative bacteria (G^−^) (Leckie, 2005; Chen et al., 2017); and 10Me16:0, 10Me17:0, 10Me17:1ω7c, and 10Me 18:0 were used to represent actinomycetes (Ac) (Zhang et al., 2022). The sum of G^+^, G^−^, Ac, and non-specific bacteria was used as the total bacteria. In addition, 18:1ω9c, 18:2ω6c, 18:3ω6c, and 16:1ω5c were chosen to represent the fungi (Joergensen and Wichern, 2008; Chang et al., 2021). The total PLFAs were the sum of bacterial and fungal PLFAs, and the B:F ratio was calculated as the bacterial-to-fungal PLFA ratio.

The structure of the microbial community was analyzed using the principal component analysis (PCA) based on the relative molar abundances of the entire PLFA signature (mol-% of the 29 most abundant FAs) after standardizing to unit variance. The PC1 scores for the entire PLFAs were used as indicators for the phylum-level microbial community structure (Chen et al., 2013).

### 2.8 Soil inorganic N ionic exchange membranes

Soil N availability was measured *in situ* using ionic exchange membranes (IEMs), which have been shown to be accurate in measuring soil N availability and generate minimal disturbance to the soil microbial community (Liu et al., 2018). We inserted one anion and one cation IEM (2.5 cm × 10 cm) in each sampling quadrat to absorb ammonium ions (NH_4_^+^) and nitrate ions (NO_3_^−^). Approximately 15 days later, we collected IEMs and immediately rinsed them with deionized water to remove soil. A pair of IEMs were placed into 150-mL flasks for extraction with 70 mL of 2 M KCl by orbital shaking (1 h at 160 rpm). NH_4_^+^ and NO_3_^−^ concentrations were analyzed using a continuous flow analyzer (AA3, Seal Analytical, Norderstedt, Germany). Soil ammonium and nitrate availability were calculated using the following formula: [(concentration in μg N per mL) × (70 mL KCl)]/(50 cm^2^ area of the strip × days in the ground) (Liu et al., 2018). Soil N availability was determined as the sum of soil ammonium and nitrate availability from the IEMs.

### 2.9 Statistical analyses

Statistical analysis was performed with IBM SPSS 27.0.1 (SPSS Inc., Chicago, IL, United States). All variables of the data were tested for normal distribution and homogeneity of variance. The effects of winter drought, chemical fertilization, and their interaction on soil properties, microbial characteristics, enzyme activities, and soil N availability were tested using a two-way ANOVA. For each specific parameter, if the interaction between winter drought and chemical fertilization (i.e., D × F) was insignificant, we used the terms non-drought (C and F plots) vs. drought (D and DF plots) for the winter drought main effect, and non-fertilization (C and D plots) vs. fertilization (F and DF plots) for the chemical fertilization main effect; if the interaction was significant, we used the terms non-drought vs. drought in non-fertilization and fertilization and then non-fertilization vs. fertilization in non-drought and drought. At the non-fertilization level, we compared C vs. D plots, and at the fertilization level, we compared F vs. DF plots; at the non-drought level, we compared C vs. F plots, and at the drought level, we compared D vs. DF plots. The Mantel test was performed for a better understanding of the correlation between soil N availability and environmental variables (including soil properties and microbial characteristics). All results were reported as means ± standard errors, and a significance level of *p* < 0.05 was used for all analyses. The figures were plotted in GraphPad Prism 9.5.0 (GraphPad Software Inc., San Diego, CA, United States) and the ggplot2 package in R software (R Development Core Team, 2023).

## 3 Results

### 3.1 Soil microclimate conditions

**Figure 1:**
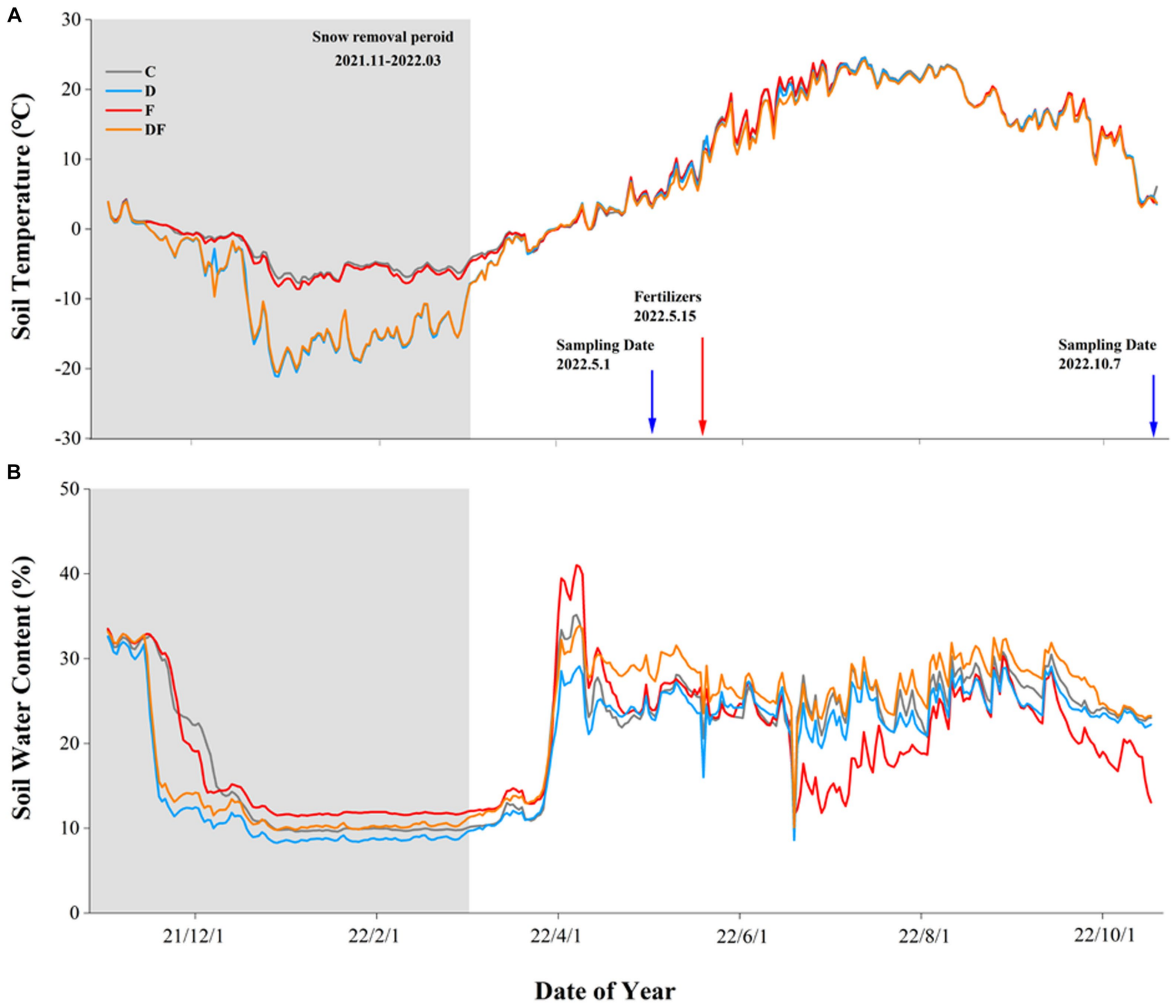
Soil temperature **(A)** and water content **(B)** in response to winter drought and chemical fertilization from November 2021 to October 2022 at 5 cm soil depth. C = control, D = winter drought, F = chemical fertilization, and DF = combined winter drought and chemical fertilization.

There was significant seasonal variation in both soil temperature and SWC, and it was described as a low temperature and dry non-growing season with a high temperature and wet growing season (Figure 1). Compared to the ambient snow plots, snow removal significantly decreased the average (−8.6 vs. −3.1°C) and minimal soil temperature (−17.0 vs. −6.8°C) at 5 cm depth over the non-growing season. Moreover, the timing of soil freezing and melting was advanced, and freeze–thaw was increased by two cycles of snow removal (Figure 1A). After 3 weeks of snowmelt, snow removal had no significant effect on SWC (Figure 1B).

### 3.2 Soil physicochemical properties

Neither winter drought nor fertilization had significant effects on SWC, DOC, DIN, and AK on the sampling dates of May and October 2022 (*p* > 0.05), but the ratio of DOC:DIN increased by 32.2% in response to the winter drought and decreased by 15.8% in response to fertilization in October. Soil pH was significantly decreased by snow removal (*p* < 0.001) but was not affected by the legacy effect of fertilization in May (*p* > 0.05). Winter drought, fertilization, and their interaction exhibited no significant impacts on the Olsen-P and DOC:Olsen-P (*p* > 0.05), but the legacy effect of fertilization profoundly decreased the DIN:Olsen-P by 27.3% in May (*p* < 0.05). Olsen-P was significantly increased by winter drought and fertilization in October (both *p* < 0.001), which caused a reduction in the DOC:Olsen-P and DIN:Olsen-P (both *p* < 0.001). In addition, a significant interactive effect of winter drought and fertilization on the DOC:Olsen-P and DIN:Olsen-P was observed (both *p* < 0.001; Table 1; Supplementary Table S1).

**Table 1:** Soil physicochemical properties in response to winter drought and chemical fertilization in May and October 2022.

Month	Treatment	SWC (%)	pH	DOC (mg kg^−1^)	DIN (mg kg^−1^)	Olsen-P (mg kg^−1^)	AK (mg kg^−1^)	DOC:DIN	DOC:Olsen-P	DIN:Olsen-P
May	C	22.3 ± 0.5	6.0 ± 0.03	183.9 ± 6.3	95.6 ± 8.9	255.5 ± 17.2	73.7 ± 4.3	2.0 ± 0.2	0.73 ± 0.06	0.37 ± 0.02
	D	22.3 ± 0.2	5.8 ± 0.02	199.9 ± 26.1	109.7 ± 8.6	274.7 ± 15.3	73.7 ± 6.2	1.9 ± 0.3	0.73 ± 0.08	0.40 ± 0.03
	F	22.9 ± 0.5	6.2 ± 0.08	251.5 ± 44.1	89.4 ± 7.1	245.2 ± 39.6	77.7 ± 1.0	2.8 ± 0.3	0.69 ± 0.06	0.28 ± 0.00
	DF	22.0 ± 0.2	5.7 ± 0.03	234.3 ± 27.8	90.4 ± 5.5	341.8 ± 40.8	66.7 ± 5.5	2.6 ± 0.4	0.71 ± 0.09	0.28 ± 0.03
October	C	24.6 ± 0.4	5.8 ± 0.11	448.4 ± 6.3	18.9 ± 2.0	10.8 ± 0.9	90.3 ± 8.8	20.8 ± 0.6	46.65 ± 0.84	1.56 ± 0.09
	D	26.6 ± 0.1	6.0 ± 0.05	402.3 ± 28.1	17.0 ± 1.4	41.2 ± 6.1	82.7 ± 6.3	28.1 ± 0.2	12.77 ± 0.21	0.42 ± 0.03
	F	24.1 ± 1.7	6.0 ± 0.06	399.3 ± 6.9	19.4 ± 2.4	44.0 ± 8.4	85.7 ± 4.2	18.0 ± 0.1	7.38 ± 0.25	0.47 ± 0.05
	DF	24.9 ± 0.5	5.9 ± 0.04	401.5 ± 20.9	19.5 ± 1.0	119.6 ± 1.9	86.3 ± 1.1	23.2 ± 0.4	3.35 ± 0.12	0.16 ± 0.01

Data are means ± 1 SE (*n* = 3).

C = control, D = winter drought, F = chemical fertilization, and DF = combined winter drought and chemical fertilization. SWC, soil water content; DOC, dissolved organic carbon; DIN, dissolved inorganic nitrogen; AK, available potassium.

### 3.3 Soil extracellular enzyme activity

The winter drought profoundly increased the activities of AG (19.9%, *p* < 0.05), CBH (72.3%, *p* < 0.05), LAP (*p* < 0.01), and AP (19.1%, *p* < 0.05) in spring sampling. Winter drought significantly increased LAP activity by 37.0% (*p* < 0.05) in non-fertilization plots, but not in fertilization plots, and a significant interactive effect of winter drought and fertilization on LAP (*p* < 0.05) was observed in May. In October, winter drought significantly increased CBH and NAG activities (both *p* < 0.05), and fertilization increased NAG activity by 179.4% (*p* < 0.01). A significant interactive effect of winter drought and fertilization on CBH activity was detected (*p* < 0.05; Table 2; Supplementary Table S2).

**Table 2:** Soil C-, N-, and P-acquiring enzyme activities in response to winter drought and chemical fertilization in May and October 2022.

Month	Treatment	AG (nmol h^−1^ g^−1^)	BG (nmol h^−1^ g^−1^)	BX (nmol h^−1^ g^−1^)	CBH (nmol h^−1^ g^−1^)	NAG (nmol h^−1^ g^−1^)	LAP (nmol h^−1^ g^−1^)	AP (nmol h^−1^ g^−1^)
May	C	13.2 ± 0.8	0.59 ± 0.09	0.57 ± 0.07	0.74 ± 0.15	0.08 ± 0.05	8.1 ± 0.1	21.6 ± 0.7
	D	15.2 ± 0.3	0.59 ± 0.03	0.93 ± 0.10	1.47 ± 0.14	0.21 ± 0.02	11.1 ± 0.6	25.5 ± 0.7
	F	11.9 ± 1.3	0.61 ± 0.04	0.63 ± 0.02	0.74 ± 0.08	0.20 ± 0.02	8.6 ± 0.3	21.9 ± 1.5
	DF	14.9 ± 0.6	0.44 ± 0.14	0.64 ± 0.17	1.08 ± 0.08	0.28 ± 0.10	9.2 ± 0.5	26.3 ± 1.6
October	C	13.6 ± 0.7	0.38 ± 0.03	0.63 ± 0.10	0.43 ± 0.08	0.13 ± 0.06	8.9 ± 0.6	24.4 ± 1.2
	D	17.6 ± 1.3	0.47 ± 0.02	0.61 ± 0.13	1.80 ± 0.30	0.21 ± 0.08	9.9 ± 0.4	28.3 ± 1.6
	F	15.2 ± 2.0	0.53 ± 0.07	0.75 ± 0.15	0.90 ± 0.16	0.26 ± 0.02	9.4 ± 1.2	25.2 ± 4.2
	DF	16.6 ± 1.3	0.63 ± 0.07	0.80 ± 0.09	0.96 ± 0.09	0.69 ± 0.09	10.0 ± 0.6	19.0 ± 1.2

Data are means ± 1 SE (*n* = 3).

C = control, D = winter drought, F = chemical fertilization, and DF = combined winter drought and chemical fertilization. C-cycling enzymes: AG, α-1,4-glucosidase; BG, β-1,4-glucosidase; BX, β-xylosidase; CBH, β-D-cellobiosidase. N-cycling enzymes: NAG, β-1,4-N-acetylglucosaminidase; LAP, L-leucine aminopeptidase. P-cycling enzyme: AP, acid phosphatase.

### 3.4 Soil microbial characteristics

**Figure 2:**
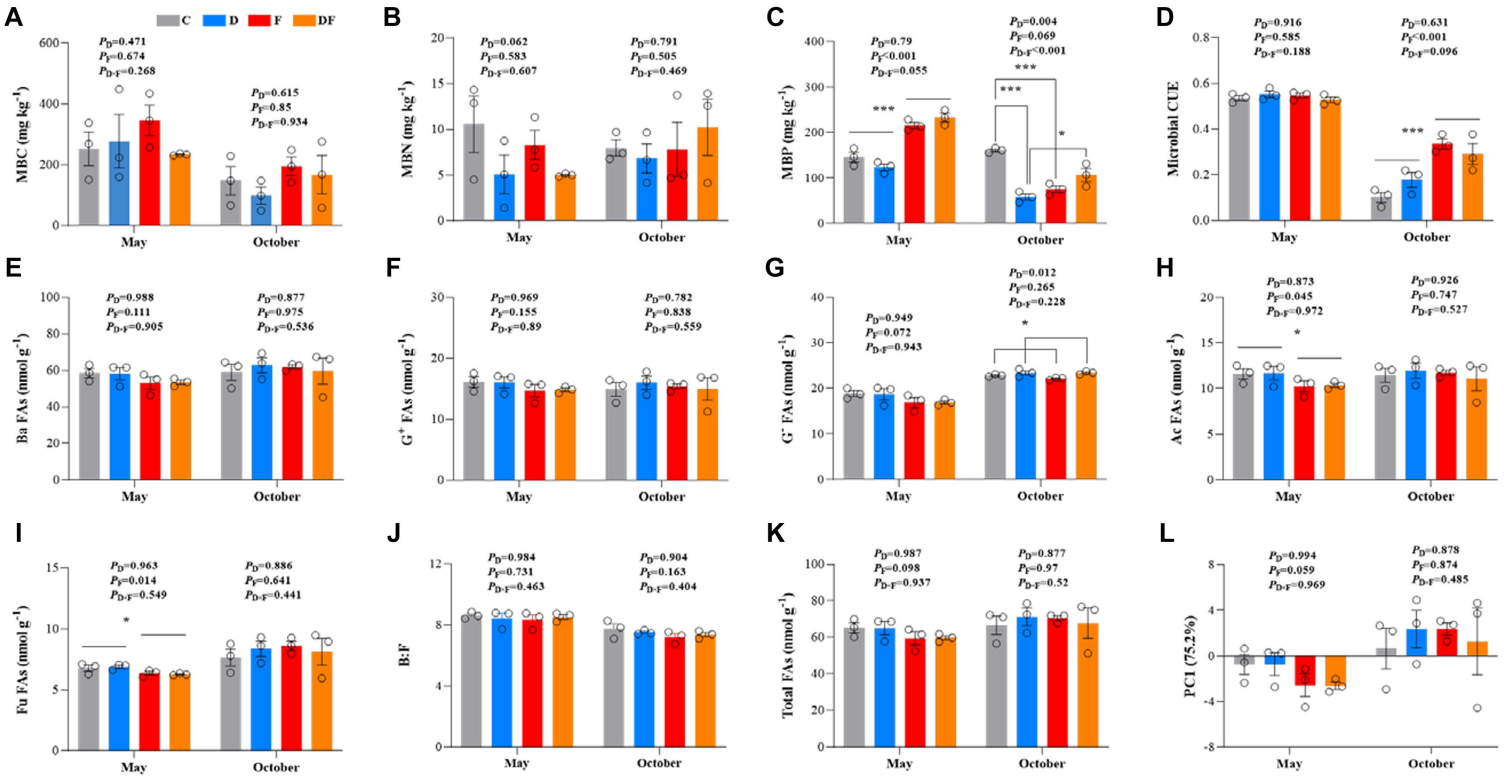
Soil microbial characteristics **(A–L)** in response to winter drought and chemical fertilization in May and October 2022. C = control, D = winter drought, F = chemical fertilization, and DF = combined winter drought and chemical fertilization. The *p*-values were expressed as follows: *p < 0.05; **p < 0.01; ***p < 0.001. Data are means ±1 SE (*n* = 3). MBC, microbial biomass carbon; MBN, microbial biomass nitrogen; MBP, microbial biomass phosphorus; CUE, microbial carbon use efficiency; Ba FAs, bacterial PLFAs; G^+^ FAs, Gram-positive PLFAs; G^−^ FAs, Gram-negative PLFAs; Ac FAs, actinomycetes PLFAs; Fu FAs, fungal PLFAs; B:F, the ratio of bacterial to fungal PLFAs.

Neither winter drought nor fertilization had significant changes in MBC or MBN (*p* > 0.05; Figures 2A,B). In May, MBP increased by 66.4% in response to the legacy effect of fertilization (*p* < 0.001). There was a significant interactive effect of winter drought and fertilization on MBP (*p* < 0.001), and winter drought significantly decreased MBP (*p* < 0.01) in October. Winter drought significantly decreased MBP by 63.9% in non-fertilization plots (*p* < 0.001) but not in **fertilization** plots (*p* > 0.05; Figure 2C). We also found that microbial CUE increased by 125% in response to fertilization in October (*p* < 0.001; Figure 2D).

Bacterial FAs, G^+^ FAs, B:F, and total FAs were not affected by winter drought, fertilization, or their interaction (*p* > 0.05; Figures 2E,F,J,K). However, there were significant declines in actinomycetes and fungal FAs due to the legacy effect of fertilization; they reduced by 11.6 and 7.3%, respectively, in May (both *p* < 0.05; Figures 2H,I). The winter drought exerted a significant positive effect on G^−^ FAs in October (increased 4.2%, *p* < 0.05; Figure 2G). No significant interactive effects of winter drought and fertilization were observed on all FAs (*p* > 0.05; Figures 2E–K). According to the PCA, there was no significant change in the phylum-level microbial community structure throughout the whole growing season by the treatments (*p* > 0.05; Figure 2L).

### 3.5 Soil N availability

**Figure 3:**
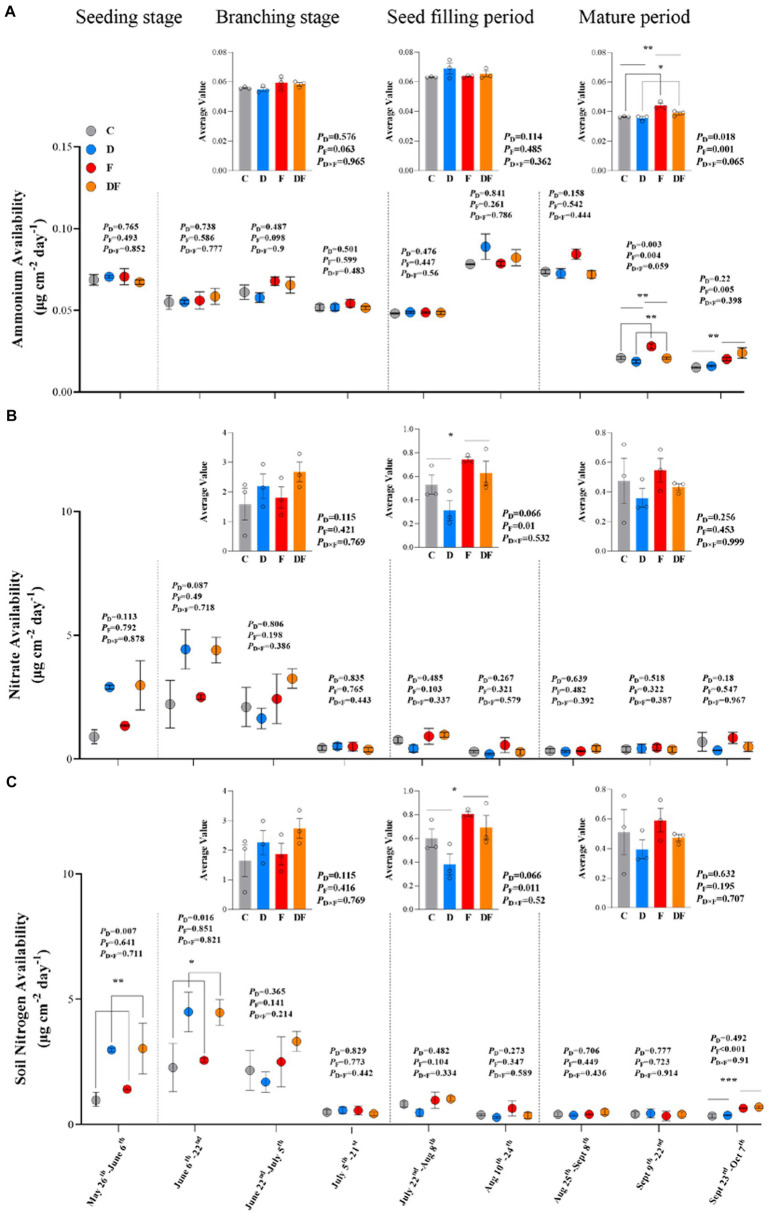
Responses of IEMs measured ammonium **(A)**, nitrate **(B)**, and N **(C)** availability to winter drought and chemical fertilization from May to October 2022. The inset figures were the changes in the average level of N availability during four growth periods of soybean. C = control, D = winter drought, F = chemical fertilization, and DF = combined winter drought and chemical fertilization. The *p*-values were expressed as follows: *p < 0.05; **p < 0.01; ***p < 0.001. Data are means ±1 SE (*n* = 3).

The IEM results showed that nitrate availability was higher than ammonium availability in this Mollisol cropland (Figures 3A,B). In spring 2022, winter drought significantly increased soil N availability (May 26–June 6 and June 6–22, *p* < 0.01 and *p* < 0.05; Figure 3C). However, as the growing season continued, the dominant treatment explaining variation in soil N availability shifted from the winter drought to fertilization (Figure 3). At the seed filling period of soybean, both nitrate and total N availability showed positive responses to fertilization (Figures 3B,C). At the mature period, ammonium availability decreased by 7.7% in response to winter drought (*p* < 0.05) and increased by 15.0% in response to fertilization (*p* < 0.01; Figure 3A). At the last sampling period (September 23–October 7), we found that fertilization significantly increased ammonium availability (*p* < 0.01; Figure 3A) and soil N availability (*p* < 0.001; Figure 3C).

### 3.6 Influencing factors of soil N availability

**Figure 4:**
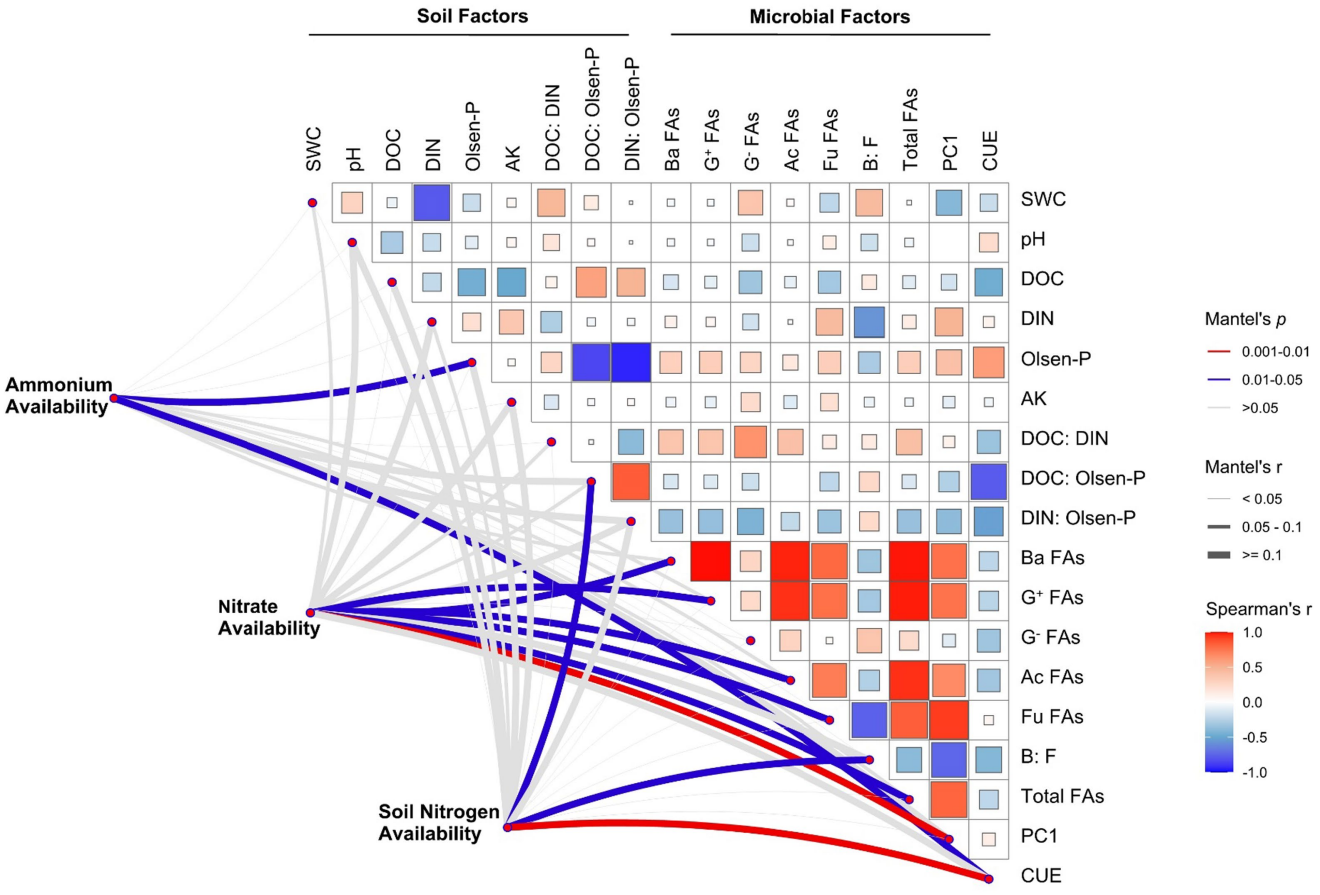
Correlations among soil N availability, soil properties, and microbial characteristics. Pairwise comparisons of soil and microbial factors are shown, with color gradients denoting Spearman’s correlation coefficients. Edge width corresponds to Mantel’s r statistic for the corresponding distance correlations. SWC, soil water content; DOC, dissolved organic carbon; DIN, dissolved inorganic nitrogen; AK, available potassium; Ba FAs, bacterial PLFAs; G^+^ FAs, Gram-positive PLFAs; G^−^ FAs, Gram-negative PLFAs; Ac FAs, actinomycetes PLFAs; Fu FAs, fungal PLFAs; B:F, the ratio of bacterial to fungal PLFAs; CUE, microbial carbon use efficiency.

The Mantel test and Spearman correlation analysis showed that ammonium availability was significantly correlated with Olsen-P content and microbial CUE (*p* < 0.05). Microbial factors explained the largest fraction of the variation in nitrate availability; the microbial community structure (PC1) exhibited the strongest correlation (*p* < 0.01), followed by Ba FAs, G^+^ FAs, Ac FAs, Fu FAs, and total FAs (all *p* < 0.05), and we found significant positive correlations among these microbial factors. Soil N availability is significantly related to microbial CUE (*p* < 0.01), DOC:Olsen-P (*p* < 0.05), and soil B:F (*p* < 0.05; Figure 4).

## 4 Discussion

### 4.1 Soil properties in response to winter drought and chemical fertilization

A number of studies have shown that winter drought could significantly increase soil C, N, and P availability, because an increase in soil freeze–thaw that may promote soil shrinkage and expansion, which gave a direct release of mineralizable organic matters through the fragmentation of soil aggregate and mortality of fine roots and microorganisms (Song et al., 2017; Ji et al., 2022). However, our results showed that soil available C, N, and P remained unchanged after the winter drought (Table 1; Supplementary Table S1). This finding might be attributed to the energy and nutrients absorbed by soil microorganisms in early spring. Watanabe et al. (2019) observed that the concentration of DIN was higher by the treatment than that in control soil after snowmelt, and then, it would gradually decrease over time. This aspect suggested that the winter drought-induced changes in the available nutrients in the soil would dissipate as air and soil temperatures increased in early spring.

Our results indicated that the available C:N:P imbalances increased in the late growing season compared with the early growing season (Table 1; Supplementary Table S1) due to the rapid increase in soil Olsen-P content. We also found that the soil available C:P and N:P ratios (DOC:Olsen-P and DIN:Olsen-P) showed a profound decline by the winter drought in October. The explanation could be due to decreased microbial immobilization (Gao et al., 2021), which was supported by our results showing that winter drought significantly decreased soil MBP (Figure 2C). Soil available C:N, C:P, and N:P ratios exhibited generally consistent responses (reduced) to chemical fertilization in October because chemical fertilization can increase the input of N and P to the soils (Zhang et al., 2019).

### 4.2 Microbial characteristics in response to winter drought and chemical fertilization

Soil EEAs are usually related to microbial metabolic rates and are widely used to indicate microbial C, N, and P requirements (Sun et al., 2023). It has been reported that soil microorganisms suffer from relative P limitation in Mollisol croplands, and microbial biomass is the key controller for microbial N/P limitation (Yang et al., 2023). In unfertilized plots, MBP was significantly decreased by winter drought at the end of the growing season (Figure 2C), which suggested that the decreased MBP may further exacerbate the microbial P limitation in this Mollisol cropland. Although soil C-, N-, and P-acquiring EEAs were increased in response to winter drought in spring, they were unchanged and remained coupled under the four treatments at the end of the growing season (Table 2; Supplementary Table S2). After the snow removal period, microbial activity was stimulated by dramatic changes in soil temperature in the spring, but the response would be attenuated as the temperature differences decreased among treatments in October.

Microbial CUE is a pivotal index for understanding soil C turnover that is driven by microbial metabolism (Tao et al., 2023). Our results showed that chemical fertilization profoundly increased microbial CUE (Figure 2D). To achieve microbial elemental homeostasis when the soil nutrient (N and P) content was sufficient through fertilization, microorganisms would devote more energy (C) for self-growth and reproduction (Li et al., 2019; Tang et al., 2020). A high soil nutrient content can also alter intracellular C partition, which leads to a lower allocation of C to respiration and a higher allocation of C to growth (Spohn et al., 2016), resulting in an increase in microbial CUE.

However, microbial community composition (bacteria, fungi, and actinomycetes) and structure (phylum level) were not sensitive to both winter drought and chemical fertilization (Figures 2E–L). It is expected that the declines in Fu FAs and Ac FAs in response to the legacy effect of chemical fertilization were detected in the early growing season (Figures 2H,I). In the studied rotation system, the former crop was maize, and it did not have the ability to recruit N-fixers; its N requirement came from N fertilization (Breza et al., 2023). To some extent, crops and microorganisms have a competitive relationship. The high N uptake capacity of maize may lead to the death of fungi and actinobacteria by N deficiency (Liu et al., 2020).

### 4.3 Soil N availability in response to winter drought and chemical fertilization

Soil ammonium availability was lower than nitrate availability during the growing season (Figures 3A,B). This finding might be due to the lower energetic cost of NH_4_^+^, which can be preferentially taken up by the plants (Legay et al., 2020). Inconsistent with our first hypothesis, soil N availability was enhanced by winter drought in the early growing season (Figure 3C). This finding suggested that winter drought-induced soil freezing may increase the mortality of microorganisms, resulting in a large amount of available N in soils (Yang et al., 2020; Ji et al., 2022). Moreover, during the soil thawing process, the surviving organisms may use dead cells as substrates to increase mineralization (Yang et al., 2023). The increased soil N availability is likely to be transient and limited to the early growing season as we did not find evidence of continued soil N accumulation during the peak growing season. This finding was consistent with that of a previous study in a northern hardwood forest, indicating that the labile N from the winter period was insufficient for the demands of both microorganisms and plants in early summer (Watanabe et al., 2019).

**Figure 5:**
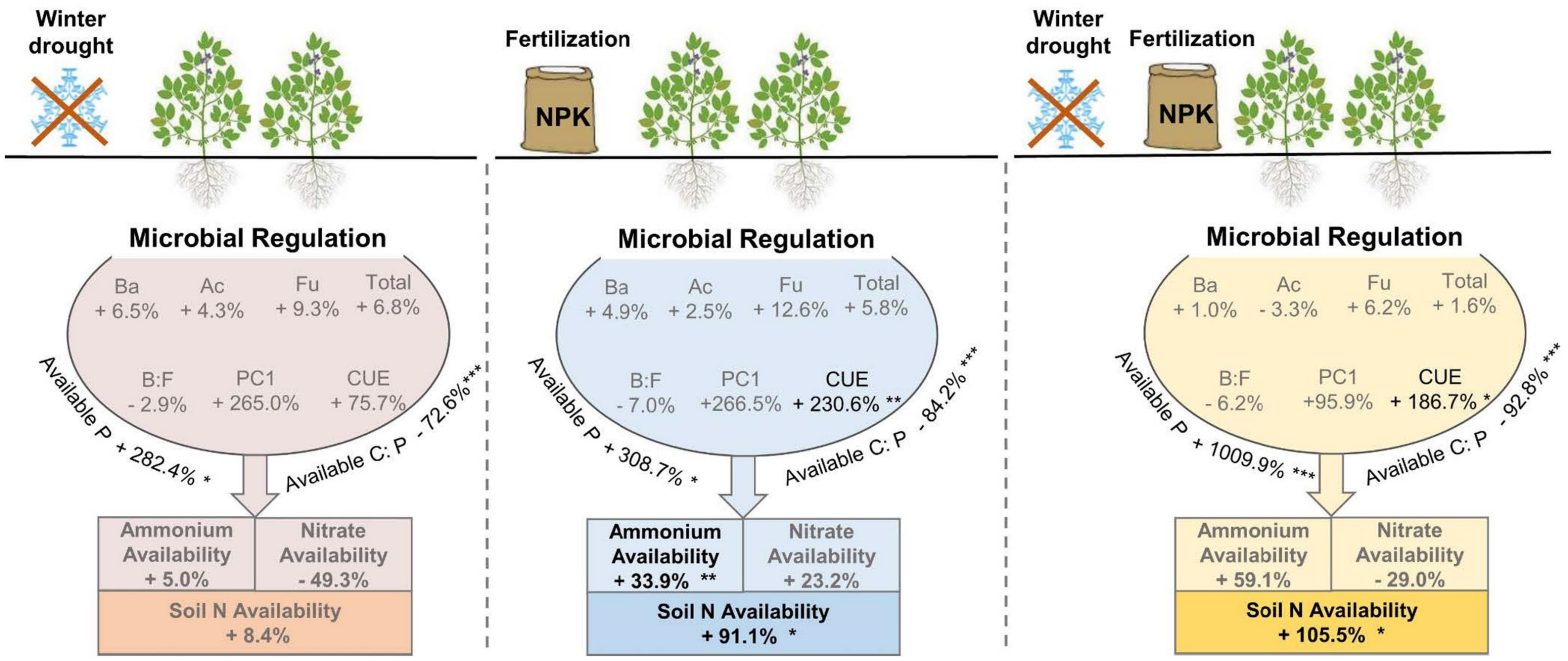
A conceptual diagram showing influences of winter drought and chemical fertilization on soil N availability. Plus and minus signs indicate the increased and decreased percentages of the response variables by winter drought, chemical fertilization, and a combination of winter drought and chemical fertilization, relative to the control plots. The *p*-values were expressed as follows: *p < 0.05; **p < 0.01; ***p < 0.001.

By contrast to the winter drought, chemical fertilization had a longer duration to affect soil N availability. During the seed filling and mature period, to some extent, soil ammonium, nitrate, and total N availability showed positive responses to chemical fertilization (Figure 3). First, fertilization was applied in the early growing season, and most of the N-fertilization was dissociated to inorganic N (i.e., NO_3_^−^ and NH_4_^+^) by soil microorganisms in late growing season. Second, plants have a low N requirement during the late growing season, which would be beneficial to the growth and/or activity of soil microorganisms, and further promote the decomposition of organic matter to increase the N availability in soils (Sun et al., 2021). We also found that a combination of winter drought and fertilization has the highest soil N availability (Figures 3C, 5). This finding agrees with a previous study that showed that combined freeze–thaw and fertilization had a larger N mineralization than single fertilization (Lin and Hernandez-Ramirez, 2022).

### 4.4 Relationships among soil properties, microbial characteristics, and soil N availability

In the present study, Olsen-P content, DOC:Olsen-P, and microbial CUE were significantly related to soil ammonium and total N availability (Figures 4, 5). A recent study conducted in a grassland ecosystem showed that soil available P played a key role in determining the growth and distribution of N-cycling microorganisms, which may improve soil N-fixing potential and increase soil N availability (Xiao et al., 2020). A large body of studies leads us to expect nutrient cycling to be coupled across space and time, especially in the N and P fertilization experiments (Achat et al., 2016). Both winter drought and chemical fertilization significantly increased Olsen-P content, which was a prerequisite for exacerbated soil ammonium and total N availability. The mineralization process releases N in the form of ammonium, which increases the amount of available N in soils.

The process of soil N mineralization has a high energy demand, and microorganisms may use soil available C substrates as an energy source to enable nitrogenase production (Schleuss et al., 2021). Our result showed that soil N availability was strongly correlated with microbial CUE under winter drought and fertilization conditions (Figures 4, 5). Multiple studies have revealed that soil microorganisms with a high CUE may reduce their respiration rate to increase their mineralization rate (Mehnaz et al., 2019; Yuan et al., 2019). Moreover, a high microbial CUE inherently requires more N to maintain the C:N ratio of their biomass (Feng et al., 2022; Zhu et al., 2023). The more assimilation of C in microbial biomass ultimately leads to a higher availability of N produced by microorganisms (Bei et al., 2022) due to the fact that N is generally coupled with C in the soils (Luo et al., 2015). The positive links between microbial CUE and soil N availability have been reported for agricultural soils with different fertilizer regimes (Spohn et al., 2016) and those were also found in unmanaged ecosystems (Tian et al., 2023).

Soil microbial biomass, structure, and composition can, directly and indirectly, influence soil nitrification and nitrate availability (Figure 4; Elrys et al., 2021; Van Huynh et al., 2023). Elrys et al. (2021) revealed that the high nitrification rates and a low soil C:N ratio caused by fertilization were associated with bacteria-dominated communities, and microbial communities produced by the nitrification process always belong to the bacteria genus (Shen et al., 2021). Additionally, although the bacteria:fungi ratio was unchanged by the winter drought and chemical fertilization, our results showed that soil N availability was related to the bacteria:fungi ratio (Figure 4). The fungal community is essential for decomposing cellulose, hemicellulose, and recalcitrant compounds, such as lignin (Shipley and Tardif, 2021). Chen et al. (2021) reported that fungi can drive soil ammonification by regulating fungal ligninolytic capacity, and changes in fungal ligninolytic capacity will, in turn, influence the release of available N. Soil N transformation processes were regulated by different microbial communities, and our results suggested that even a slight change in the ratio of bacteria to fungi would alter soil N availability.

## 5 Conclusion

In conclusion, our results showed that the effect of chemical fertilization rather than winter drought on soil N availability can continue throughout the growing season in agroecosystems. Chemical fertilization significantly increased microbial CUE and soil N availability, and a combination of winter drought and chemical fertilization had the highest soil N availability at the end of the growing season. Both microbial and soil properties have strong linkages with soil N availability. Although the microbial biomass and extracellular enzyme activities had quick responses to winter drought and chemical fertilization, the durations were less than a growing season. We found that microbial CUE was the most positive correlation factor with soil N availability, followed by soil available C:P and bacteria:fungi ratios. We concluded that the relationship between soil microbial function and N availability was crucial for understanding soil N-cycling processes under global change and agricultural management practices. We also highlighted the need to explicitly incorporate microbial characteristics into biogeochemical models to improve the prediction of ecosystem N cycling.

## Data availability statement

The raw data supporting the conclusions of this article will be made available by the authors, without undue reservation.

## Author contributions

WB: Data curation, Formal analysis, Investigation, Visualization, Writing – original draft, Writing – review & editing. PH: Investigation, Writing – review & editing. LH: Investigation, Writing – review & editing. XW: Writing – review & editing. LF: Investigation, Writing – review & editing. JZ: Funding acquisition, Writing – review & editing. JW: Funding acquisition, Writing – review & editing. XY: Conceptualization, Data curation, Formal analysis, Funding acquisition, Investigation, Project administration, Visualization, Writing – original draft, Writing – review & editing. L-JL: Conceptualization, Funding acquisition, Project administration, Writing – review & editing.
