# Supplementary material for: Soil nitrogen availability and microbial carbon use efficiency are dependent more on chemical fertilization than winter drought in a maize–soybean rotation system
Source: Front Microbiol. 2024 Mar 14;15:1304985. doi: 10.3389/fmicb.2024.1304985 (PMC10972866; doi:10.3389/fmicb.2024.1304985)
Supplement: Supplementary file 1 [file Table_1.docx]

**Supporting Information**

**Soil nitrogen availability and microbial carbon use efficiency are dependent more on chemical fertilization than winter drought in a maize–soybean rotation system**

***Corresponding authors**

**Xuechen Yang** E-mail: [yangxuechen@iga.ac.cn](mailto:yangxuechen@iga.ac.cn)

Northeast Institute of Geography and Agroecology, Chinese Academy of Sciences

138 Haping Road, Harbin, Heilongjiang 150081, P. R. China

**Jihua Wang**  E-mail: [wangjihua333@hotmail.com](mailto:wangjihua333@hotmail.com)

Harbin Normal University

1 Shida Road, Harbin, Heilongjiang 150025, P. R. China

**STable 1.** Results (*F* values) of the two-way ANOVA for the effects of winter drought, chemical fertilization, and their interactions on soil physicochemical properties in May and October 2022. The *P* values were expressed as follows: *, *P* < 0.05; **, *P* < 0.01; ***, *P* < 0.001.

| **Month** | **Treatment** | **SWC**  **(%)** | **pH** | **DOC**  **(mg kg^−1^)** | **DIN**  **(mg kg^−1^)** | **Olsen-P**  **(mg kg^−1^)** | **AK**  **(mg kg^−1^)** | **DOC: DIN** | **DOC: Olsen-P** | **DIN: Olsen-P** |
| --- | --- | --- | --- | --- | --- | --- | --- | --- | --- | --- |
| **May** | **D** | 0.971 | 37.673******* | 0.000 | 0.654 | 2.374 | 0.921 | 0.083 | 0.005 | 0.203 |
|  | **F** | 0.140 | 1.507 | 2.022 | 1.853 | 0.572 | 0.068 | 4.499 | 0.115 | 17.484***** |
|  | **D×F** | 0.845 | 4.584 | 0.213 | 0.487 | 1.064 | 0.921 | 0.001 | 0.013 | 0.325 |
|  |  |  |  |  |  |  |  |  |  |  |
| **October** | **D** | 1.534 | 0.235 | 0.000 | 0.176 | 66.870******* | 0.241 | 179.244******* | 1151.924******* | 127.417******* |
|  | **F** | 0.824 | 0.018 | 0.020 | 0.435 | 74.200******* | 0.005 | 66.744******* | 1901.361******* | 112.568******* |
|  | **D×F** | 0.249 | 2.717 | 0.017 | 0.219 | 12.123****** | 0.342 | 5.206 | 714.628******* | 42.543******* |

D = winter drought, F = chemical fertilization, and D**×**F = the interaction of winter drought and chemical fertilization.

Abbreviations: SWC, soil water content; DOC, dissolved organic carbon; DIN, dissolved inorganic N (NH_4_^+^-N and NO_3_^−^-N); AK, available potassium.

**STable 2.** Results (*F* values) of the two-way ANOVA for the effects of winter drought, chemical fertilization, and their interactions on soil C-, N-, and P-acquiring enzyme activities in May and October 2022. The *P* values were expressed as follows: *, *P* < 0.05; **, *P* < 0.01; ***, *P* < 0.001.

| **Month** | **Treatment** | **AG**  **(nmol h^−1^g^−1^)** | **BG**  **(nmol h^−1^g^−1^)** | **BX**  **(nmol h^−1^g^−1^)** | **CBH**  **(nmol h^−1^g^−1^)** | **NAG**  **(nmol h^−1^g^−1^)** | **LAP**  **(nmol h^−1^g^−1^)** | **AP**  **(nmol h^−1^g^−1^)** |
| --- | --- | --- | --- | --- | --- | --- | --- | --- |
| **May** | **D** | 6.277***** | 0.648 | 2.064 | 14.065***** | 2.336 | 11.878****** | 7.950***** |
|  | **F** | 0.651 | 0.357 | 0.795 | 1.866 | 1.666 | 1.512 | 0.135 |
|  | **D×F** | 0.195 | 0.746 | 1.860 | 1.898 | 0.122 | 5.331***** | 0.023 |
|  |  |  |  |  |  |  |  |  |
| **October** | **D** | 2.298 | 2.075 | 0.018 | 10.663***** | 9.049***** | 0.84 | 1.769 |
|  | **F** | 0.033 | 5.222 | 1.084 | 0.709 | 13.572****** | 0.100 | 0.059 |
|  | **D×F** | 0.552 | 0.010 | 0.043 | 8.960***** | 4.328 | 0.030 | 0.001 |

D = winter drought, F = chemical fertilization, and D**×**F = the interaction of winter drought and chemical fertilization.

Abbreviations: C-cycling enzymes: AG, α-1,4-glucosidase; BG, β-1,4-glucosidase; BX, β-xylosidase; CBH, β-D-cellobiosidase. N-cycling enzymes: NAG, β-1,4-N-acetylglucosaminidase; LAP, L-leucine aminopeptidase. P-cycling enzyme: AP, acid phosphate.
